# Women, men, and rheumatoid arthritis: analyses of disease activity, disease characteristics, and treatments in the QUEST-RA Study

**DOI:** 10.1186/ar2591

**Published:** 2009-01-14

**Authors:** Tuulikki Sokka, Sergio Toloza, Maurizio Cutolo, Hannu Kautiainen, Heidi Makinen, Feride Gogus, Vlado Skakic, Humeira Badsha, Tõnu Peets, Asta Baranauskaite, Pál Géher, Ilona Újfalussy, Fotini N Skopouli, Maria Mavrommati, Rieke Alten, Christof Pohl, Jean Sibilia, Andrea Stancati, Fausto Salaffi, Wojciech Romanowski, Danuta Zarowny-Wierzbinska, Dan Henrohn, Barry Bresnihan, Patricia Minnock, Lene Surland Knudsen, Johannes WG Jacobs, Jaime Calvo-Alen, Juris Lazovskis, Geraldo da Rocha Castelar Pinheiro, Dmitry Karateev, Daina Andersone, Sylejman Rexhepi, Yusuf Yazici, Theodore Pincus, the QUEST-RA Group

**Affiliations:** 1 Jyväskylä Central Hospital, Keskussairaalantie 19, 40620 Jyväskylä, and Medcare Oy, Hämeentie 1, 44100 Äänekoski, Finland; 2 Division of Rheumatology, Hospital San Juan Bautista, Avenida Illia 200, Catamarca, CP:4700, Argentina; 3 Research Laboratories and Clinical Academic Unit of Rheumatology, University of Genova Italy, Viale Benedetto XV, 6, 16132 Genova, Italy; 4 Medcare Oy, Hämeentie 1, 44100 Äänekoski, Finland; 5 Jyväskylä Central Hospital, Keskussairaalantie 19, 40620 Jyväskylä, Finland; 6 Gazi University, Department of Physical Medicine and Rehabilitation, Division of Rheumatology, 06530 Ankara, Turkey; 7 Rheumatology Department, Institute of Rheumatology 'Niska Banja', Srpskih junaka 1, Nis, 18205 Serbia; 8 Rheumatology Department, Dubai Bone and Joint Center, Al Razi Building, DHCC, PO Box 118855, Dubai 118855, United Arab Emirates; 9 Rheumatology Department, East-Tallinn Central Hospital, Pärnu Road 104, Tallinn 11312, Estonia; 10 Rheumatology Department, Kaunas University of Medicine, Eiveniu str.2, Kaunas LT50009, Lithuania; 11 1st Department of Rheumatology, Hospitaller Brothers of St John of God Budapest, Árpád f.u.7, H-1027, Budapest, Hungary; 12 National Health Center Dept. of Rheumatology, Podmaniczky u. 72, H-1063, Budapest, Hungary; 13 Department of Dietetics and Nutrition Science, Harokopio University of Athens and Department of Internal Medicine and Clinical Immunology, Euroclinic of Athens, Athanasiadou 9, 11521, Athens, Greece; 14 Department of Internal Medicine and Clinical Immunology, Euroclinic of Athens, Athanasiadou 9, 11521, Athens, Greece; 15 Department of Internal Medicine II, Rheumatology, Schlosspark-Klinik Teaching Hospital of the Charité, University Medicine Berlin, Heubnerweg 2, 14059 Berlin, Germany; 16 Service de Rhumatologie, CHU de Strasbourg, Hôpital Hautepierre, Avenue Molière, BP 49, 67098 Strasbourg, France; 17 Department of Rheumatology, Polytechnic University of Marche, Via dei Colli, 52, 60035, Jesi, Ancona, Italy; 18 Poznan Rheumatology Center in Srem, 95 Mickiewicz Street, 63-100 Srem, Poland; 19 Wojewodzki Zespol Reumatologiczny im. dr Jadwigi Titz-Kosko, Ul. Grunwaldzka 1/3, 81-759 Sopot, Poland; 20 Department of Rheumatology, Uppsala University Hospital, S-75185, Uppsala, Sweden; 21 Rheumatology Rehabilitation, Our Lady's Hospice and St. Vincent's University Hospital, Elm Park, Dublin, and University College, Dublin, Ireland; 22 Rheumatology Rehabilitation, Our Lady's Hospice, Harold's Cross, Dublin, Ireland; 23 Rheumatology Department, Copenhagen University Hospital at Herlev, Herlev Ringvej 75, 2730 Herlev, Denmark; 24 Department of Rheumatology and Clinical Immunology F02.127, University Medical Center Utrecht, P.O. Box 85500, 3508 GA Utrecht, The Netherlands; 25 Rheumatology Division, Hospital General Sierrallana, Av. M. Teira s/n 39300 Torrelavega, Cantabria, Spain; 26 Rheumatology Section, Riverside Professional Center, 31 Riverside Drive, Sydney, NS, B1S 3N1, Canada; 27 Internal Medicine, Pedro Ernesto University Hospital, Boulevard 28 de Setembro 77 sala 333, Rio de Janeiro, 20551-030, Brazil; 28 Department of Early Arthritis, Institute of Rheumatology, Kashirskoye shosse, 34a, Moscow, 115522, Russia; 29 Medical Faculty of Latvia University, P. Stradina Clinical University Hospital, Pilsonu Street 13, LV 1002, Riga, Latvia; 30 Rheumatology Department, University Clinical Center of Kosova, Kodra e diellit, Rr. II, Lamela 11/9, Prishtina, 10 000, Kosova; 31 New York University Hospital for Joint Diseases, 301 East 17 Street, New York, NY 10003, USA

## Abstract

**Introduction:**

Gender as a predictor of outcomes of rheumatoid arthritis (RA) has evoked considerable interest over the decades. Historically, there is no consensus whether RA is worse in females or males. Recent reports suggest that females are less likely than males to achieve remission. Therefore, we aimed to study possible associations of gender and disease activity, disease characteristics, and treatments of RA in a large multinational cross-sectional cohort of patients with RA called Quantitative Standard Monitoring of Patients with RA (QUEST-RA).

**Methods:**

The cohort includes clinical and questionnaire data from patients who were seen in usual care, including 6,004 patients at 70 sites in 25 countries as of April 2008. Gender differences were analyzed for American College of Rheumatology Core Data Set measures of disease activity, DAS28 (disease activity score using 28 joint counts), fatigue, the presence of rheumatoid factor, nodules and erosions, and the current use of prednisone, methotrexate, and biologic agents.

**Results:**

Women had poorer scores than men in all Core Data Set measures. The mean values for females and males were swollen joint count-28 (SJC28) of 4.5 versus 3.8, tender joint count-28 of 6.9 versus 5.4, erythrocyte sedimentation rate of 30 versus 26, Health Assessment Questionnaire of 1.1 versus 0.8, visual analog scales for physician global estimate of 3.0 versus 2.5, pain of 4.3 versus 3.6, patient global status of 4.2 versus 3.7, DAS28 of 4.3 versus 3.8, and fatigue of 4.6 versus 3.7 (*P *< 0.001). However, effect sizes were small-medium and smallest (0.13) for SJC28. Among patients who had no or minimal disease activity (0 to 1) on SJC28, women had statistically significantly higher mean values compared with men in all other disease activity measures (*P *< 0.001) and met DAS28 remission less often than men. Rheumatoid factor was equally prevalent among genders. Men had nodules more often than women. Women had erosions more often than men, but the statistical significance was marginal. Similar proportions of females and males were taking different therapies.

**Conclusions:**

In this large multinational cohort, RA disease activity measures appear to be worse in women than in men. However, most of the gender differences in RA disease activity may originate from the measures of disease activity rather than from RA disease activity itself.

## Introduction

The possible influence of gender and gender-related variables on the phenotype, severity, and prognosis of rheumatoid arthritis (RA) appears to be of considerable interest [1]. Severe clinical disease activity, structural damage, and deformities have been reported equally in both genders in RA [2-6]. Generally, however, women report more severe symptoms [7] and greater disability [8] and often have higher work disability rates [9] compared with men. As in the general population, men with RA have considerably higher mortality rates than women [10]. However, the clinical status of RA patients at this time is improved compared with previous decades, according to disease activity [11,12] and function and structural outcomes [12-17], generally with no gender differences.

Analyses of gender differences of RA include a study that indicates less favorable status in men [18] and many studies with less favorable status in women [19-23]. Some recent studies suggest that men have better responses to treatments with biologic agents than women [19-21], and other studies indicate that male gender is a major predictor of remission in early RA [22,23]. However, men have been shown to experience a greater number of adverse effects, particularly serious infections during biologic treatments [24,25]. Similar treatment goals have been advocated for both genders [26].

Gender differences in disease activity and other measures may reflect the properties of measures [27] as females have higher erythrocyte sedimentation rates (ESRs) than males [28] and poorer scores on most questionnaires [7]. Further information concerning the possible influence of gender on the clinical status and disease activity measures of RA appears to be of value. Therefore, we explored possible associations of gender and disease activity measures, treatments, and clinical characteristics of RA in a large multinational cross-sectional cohort of patients with RA [29], as presented in this report.

## Materials and methods

The Quantitative Standard Monitoring of Patients with RA (QUEST-RA) program was established in 2005 to promote quantitative assessment in usual clinical care at multiple sites and to develop a database of RA patients seen outside of clinical trials in regular care in many countries. The initial design was to assess 100 patients with RA at each of three or more sites in 10 different countries, with data collection beginning in January 2005. The program has since been expanded to include 6,004 patients from 70 sites in 25 countries as of April 2008. This report [29] includes data from Argentina, Brazil, Canada, Denmark, Estonia, Finland, France, Germany, Greece, Hungary, Ireland, Italy, Kosovo, Latvia, Lithuania, The Netherlands, Poland, Russia, Serbia, Spain, Sweden, Turkey, United Arab Emirates, the UK, and the US. The study was carried out in compliance with the Declaration of Helsinki. Ethics committees or internal review boards of participating institutes approved the study, and informed consent was obtained from the patients.

### Clinical evaluation

All patients were assessed according to a standard protocol to evaluate RA (SPERA) [30]. Physicians completed three one-page forms: (a) review of clinical features, including classification criteria, extra-articular features, comorbidities, and relevant surgeries; (b) all previous and present disease-modifying antirheumatic drugs (DMARDs), their adverse events, and reasons for discontinuation; and (c) a 42-joint count [31] for swollen and tender joints as well as joints with limited motion or deformity. The review included physician global assessment of disease activity, physician report concerning whether or not the patient had radiographic erosions, and laboratory tests of ESR, C-reactive protein (CRP), and rheumatoid factor (RF) values.

### Patient self-report

Patients completed a four-page expanded self-report questionnaire that included the standard Health Assessment Questionnaire (HAQ) [32] as well as items from the multidimensional HAQ (MDHAQ) [33], HAQ II [34], and the Recent-Onset Arthritis Disability questionnaire to assess functional capacity in activities of daily living [35]. The questionnaire also includes visual analog scales (VASs) for pain, patient global status, and fatigue; RA disease activity index (RADAI) self-report joint count [36]; duration of morning stiffness; lifestyle choices such as smoking and physical exercise; height and weight to calculate body mass index; and demographic data, including years of education and work status [29].

### Gender and disease activity measures

DAS28 was calculated for current disease activity [37,38] according to 28 swollen (SJC28) and tender (TJC28) joint counts from the formula DAS28 = 0.56*sqrt(TJC28) + 0.28*sqrt(SJC28) + 0.70*Ln(ESR) + 0.014*patient global 0 to 100. DAS28 remission rates (DAS28 of less than 2.6) were calculated for females and males. The HAQ score was calculated without including 'aids and devices' and 'help from other people' given that the availability of aids/devices may differ across countries in a multicultural study such as QUEST-RA. It has been suggested that the HAQ may be calculated without aids/devices/help since inclusion might result in bias.

The proportion of patients who met DAS28 criteria for remission (<2.6) was analyzed in females and males with 0, 1, 2, 3, 4, and 5 swollen joints on a 28-joint count. Levels of individual disease activity measures were calculated for females and males according to SJC28 in arbitrary categories of 0 to 1, 2 to 3, 4 to 6, and 7 or more swollen joints. Swollen joint count was chosen as the standard, although a 'gold standard' measure for disease activity does not exist.

### Gender and disease characteristics

Gender differences in disease characteristics, including the prevalence of RF^+^, nodules, and erosive disease, were studied separately in two groups of countries: 'low' prevalence and 'high' prevalence countries. For example, the prevalence of RF^+ ^ranges between 52% and 92% among countries, with a median of 73.5%. Thus, all countries with a 'low' prevalence of RF^+ ^of between 52% and 73.5% were analyzed together for gender differences of RF^+^, and countries with a 'high' prevalence of RF^+ ^of between 73.5% and 92% were analyzed together for gender differences of RF^+^. Two groups of countries ('low' versus 'high' prevalence) were formed similarly to analyze nodules (cut point for prevalence = 20%) and erosive disease (cut point for prevalence = 63%).

### Gender and therapies for rheumatoid arthritis

The percentage of patients who were taking prednisone, methotrexate, and biologic agents for RA differed considerably between countries. To study whether females were treated differently from males, countries were studied in two groups, such as for disease characteristics, divided at the median among the 25 countries. Thus, countries with 'low use of a drug' and 'high use of a drug' were analyzed separately. Medians were 50% for prednisone, 62% for methotrexate, and 18% for biologic agents. The delay between the first RA symptoms and initiation of the first DMARDs was calculated and compared between men and women.

### Statistical methods

Results for continuous variables are presented as mean, standard deviation, median, and percentages. Statistical significance was tested with the Student *t *test and nonparametric tests for continuous variables and the chi-square test for categorical variables. The association of gender and outcome variables was calculated for each variable and each country. Effect size was estimated according to two methods: in standardized units of difference (Cohen's D) and variance-accounted statistics (eta-squared [η^2^]). Ninety-five percent confidence intervals for Cohen's D were obtained by bias-corrected bootstrapping (1,000 replications) and for η^2 ^by non-centrality-based interval estimation. η^2 ^was calculated using an analysis of covariance model that adjusts for age, disease duration, and country. Cohen's D standards are small effect 0.2, medium effect 0.5, and large effect 0.8. For η^2^, standards are small effect 0.01, medium effect 0.059, and large effect 0.138.

## Results

### Demographic and clinical characteristics

In April 2008, the QUEST-RA database included 6,004 patients from 70 sites in 25 countries. The demographic characteristics are those of a typical RA cohort with 79% females, more than 90% Caucasians, and a mean age of 57 years (Table 1), with considerable variation between countries. Significant variation between countries was seen in disease activity, severity, and treatments (Table 1).

**Table 1:** Patient demographic and clinical characteristics in the QUEST-RA Study by country

Country	Sites	Patients	Female, percentage	Age, years	Disease duration, years	DMARD delay, months	RF^+^, percentage	Smoking now, percentage	DAS28	SJC28	ESR	Pain	Patient global	HAQ	Taking now, percentage		
															
															Pred	MTX	Any biologic
Netherlands	3	317	66.3	59.2	9.2	6.0	68.8	22.9	3.1	1.0	15.0	2.5	2.7	0.8	16.1	74.1	19.6
Finland	3	304	72.4	58.5	13.5	7.0	74.8	15.5	3.3	1.0	13.0	2.8	2.8	0.6	51.3	61.5	12.5
USA	3	301	72.9	57.5	9.3	9.0	70.9	20.5	3.3	2.0	14.0	3.2	2.6	0.6	60.1	71.8	27.6
Greece	3	300	75.7	58.1	11.8	7.0	52.1	15.8	3.4	0.0	23.0	2.3	2.0	0.3	70.7	71.3	47.0
Denmark	3	301	76.7	57.8	12.0	11.0	73.3	31.6	3.4	1.0	14.0	2.6	2.8	0.6	14.6	71.1	21.3
Spain	3	302	73.5	59.8	10.6	14.0	72.5	17.8	3.5	1.0	17.0	3.1	3.6	0.9	46.7	56.3	23.2
France	4	389	77.9	55.3	12.8	8.0	75.3	19.1	3.7	1.0	16.0	3.9	3.6	0.9	60.9	57.1	44.2
Sweden	3	260	71.8	59.4	12.5	12.0	81.6	19.2	3.8	2.0	19.0	3.3	3.3	0.9	41.2	65.8	26.9
UK	3	145	77.9	59.6	15.0	16.0	81.4	19.4	4.0	1.0	19.0	4.1	3.6	0.9	28.3	70.3	14.5
Ireland	3	240	64.3	56.4	11.3	11.0	79.6	24.6	4.1	3.0	18.0	3.4	2.9	0.8	31.7	71.7	32.1
Canada	1	100	78.8	57.9	12.4	11.0	82.8	30.0	4.1	2.0	21.0	4.6	4.0	1.0	25.0	49.0	23.0
Turkey	3	309	85.6	52.2	11.6	12.0	67.6	14.1	4.2	0.0	30.0	4.2	4.6	0.9	57.3	69.3	5.8
Brazil	3	115	88.6	52.3	8.5	8.0	79.6	13.3	4.2	3.0	28.0	3.2	3.5	0.6	53.9	80.0	25.2
UAE	2	199	85.8	45.3	6.4	11.7	75.4	6.3	4.3	3.0	23.5	3.7	2.8	0.6	35.2	53.3	7.5
Germany	3	225	83.6	58.8	13.4	15.0	60.9	13.5	4.4	3.0	20.0	5.0	4.9	0.8	26.7	45.8	22.7
Italy	4	336	78.2	61.0	10.5	9.0	71.4	15.9	4.5	2.0	28.0	4.9	5.0	1.0	51.8	53.3	12.5
Estonia	3	168	85.5	55.8	11.8	12.0	68.1	14.0	4.7	4.0	24.0	4.3	4.8	1.1	43.5	53.6	0.6
Russia	3	73	84.5	54.2	6.2	10.0	74.6	15.4	5.0	5.0	24.0	3.6	4.5	1.1	38.4	46.6	16.4
Hungary	3	153	87.4	57.9	12.6	12.0	92.8	25.3	5.1	5.0	26.0	5.2	5.1	1.4	36.6	62.7	12.4
Latvia	1	79	79.7	53.2	13.2	32.1	84.6	19.2	5.2	4.0	26.0	5.1	5.7	1.4	63.3	70.9	21.5
Poland	7	642	86.7	53.2	11.5	4.0	70.3	11.9	5.3	6.0	31.0	5.0	4.8	1.4	58.9	65.0	6.1
Argentina	2	246	90.2	51.4	9.9	13.0	90.5	21.4	5.3	9.0	30.0	5.0	4.7	1.0	63.4	48.8	2.8
Lithuania	2	300	82.9	54.1	10.7	15.0	78.4	7.1	5.5	3.0	29.0	5.2	5.3	1.4	82.7	55.7	9.3
Serbia	1	100	88.0	59.2	10.1	11.1	71.4	18.4	5.9	6.0	28.0	5.1	5.3	1.6	54.0	54.0	0.0
Kosovo	1	100	84.0	55.0	7.8	3.0	80.6	10.4	6.0	6.0	45.0	5.1	5.0	1.6	90.0	71.0	1.0

Total	70	6,004	79.2	56.2	11.2	10.0	73.6	17.4	4.2	2.0	22.0	4.1	4.2	0.9	49.1	62.5	18.3

Mean values are presented for age, disease duration, and disease activity score using 28 joint counts (DAS28). Median values are presented for other continuous variables. DMARD, disease-modifying antirheumatic drug; ESR, erythrocyte sedimentation rate; HAQ, Health Assessment Questionnaire; MTX, methotrexate; Pred, prednisone; QUEST-RA, Quantitative Standard Monitoring of Rheumatoid Arthritis; RF, rheumatoid factor; SJC, swollen joint count; UAE, United Arab Emirates.

### Gender differences in disease activity in the entire group

**Figure 1:**
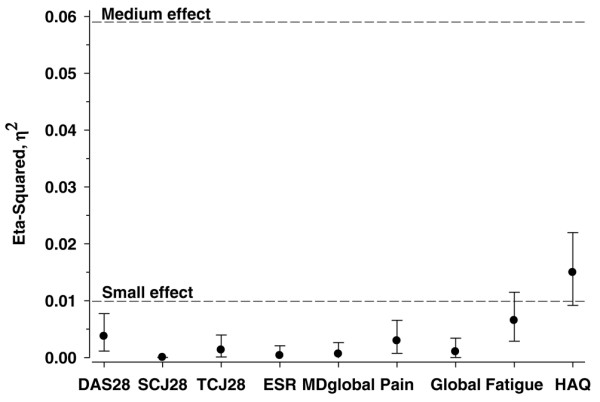
**Differences according to gender among clinical variables in the QUEST-RA Study, adjusted for age, disease duration, and country**. DAS28, disease activity score using 28 joint counts; ESR, erythrocyte sedimentation rate; HAQ, Health Assessment Questionnaire; MDglobal, doctor global assessment; QUEST-RA, Quantitative Standard Monitoring of Rheumatoid Arthritis; SJC28, swollen joint count-28; TJC28, tender joint count-28.

Women had higher scores (indicating poorer status) than men in all Core Data Set measures. The mean values for females and males were SJC28 of 4.5 versus 3.8, TJC28 of 6.9 versus 5.4, ESR of 30 versus 26, HAQ (0 to 3) of 1.1 versus 0.8, visual analog scales (0 to 10) for physician global estimate of 3.0 versus 2.5, pain of 4.3 versus 3.6, and patient global estimate of 4.2 versus 3.7 (*P *< 0.001). DAS28 (0 to 10) was 4.3 in females versus 3.8 in males, and fatigue was 4.6 versus 3.7 (*P *< 0.001). Variables were also compared using nonparametric tests, with identical levels of statistical significance. Cohen's D effect size of gender was at a medium level (0.2 to 0.5) for HAQ physical function (0.43) followed by DAS28 and fatigue (0.33), pain (0.27), physician global estimate (0.23), tender joint count (0.21), and patient global estimate (0.20) and was at a low level (<0.20) for swollen joint count and ESR (0.13 for both). According to η^2 ^statistics, the effect of gender was small-medium for all studied variables (Figure 1).

Among the disease activity measures that were studied, SJC28 levels appeared to be most similar between genders. Therefore, other disease activity measures were compared on different SJC28 levels. Among patients who had 0 to 1 swollen joints, women had statistically significantly higher mean values compared with men for all other disease activity measures (*P *< 0.001) (Table 2). Among patients who had 2 to 3 swollen joints, women had significantly higher scores than men in all other measures except TJC and ESR (Table 2). At higher levels of SJC28, differences were most pronounced in pain, fatigue, and HAQ.

**Table 2:** Differences in disease activity measures between females and males in the QUEST-RA Study according to the number of swollen joints

Number of swollen joints	Mean (standard deviation)							
	
	DAS28	TJC28	ESR	MD global	Pain	Patient global	Fatigue	HAQ
0–1								
Female	3.2 (1.2)	3.2 (5.3)	24 (19)	1.6 (1.8)	3.3 (2.6)	3.3 (2.5)	3.9 (2.9)	0.83 (0.75)
Male	2.7 (1.2)	2.0 (3.9)	20 (21)	1.2 (1.5)	2.6 (2.5)	2.9 (2.6)	2.9 (2.7)	0.52 (0.62)
*P *value	<0.001	<0.001	<0.001	<0.001	<0.001	<0.001	<0.001	<0.001
2–3								
Female	4.2 (1.1)	5.2 (5.4)	29 (22)	2.8 (1.9)	4.1 (2.6)	4.1 (2.4)	4.5 (2.8)	1.1 (0.71)
Male	3.8 (1.2)	4.7 (5.7)	25 (26)	2.4 (1.8)	3.6 (2.4)	3.6 (2.3)	3.7 (2.6)	0.74 (0.62)
*P *value	<0.001	0.26	0.11	0.013	0.010	0.010	0.001	<0.001
4–6								
Female	4.8 (1.1)	7.3 (6.0)	31 (23)	3.5 (2.0)	4.7 (2.5)	4.6 (2.4)	5.0 (2.7)	1.2 (0.71)
Male	4.6 (1.3)	6.8 (6.2)	32 (27)	3.4 (2.0)	4.1 (2.5)	4.4 (2.4)	4.2 (2.7)	0.91 (0.64)
*P *value	0.071	0.28	0.63	0.041	0.017	0.29	<0.001	<0.001
≥ 7								
Female	6.0 (1.2)	13 (8.0)	38 (25)	5.1 (2.1)	5.6 (2.5)	5.2 (2.5)	5.6 (2.6)	1.4 (0.75)
Male	5.8 (1.3)	12 (7.8)	38 (28)	4.9 (2.1)	5.2 (2.3)	4.9 (2.4)	5.0 (2.6)	1.2 (0.68)
*P *value	0.016	0.064	0.99	0.21	0.024	0.082	<0.001	<0.001

*P *values are from Student *t *test for independent samples. DAS28, disease activity score using 28 joint counts; ESR, erythrocyte sedimentation rate; HAQ, Health Assessment Questionnaire; MD global, doctor global assessment; QUEST-RA, Quantitative Standard Monitoring of Rheumatoid Arthritis; TJC28, tender joint count-28.

**Figure 2:**
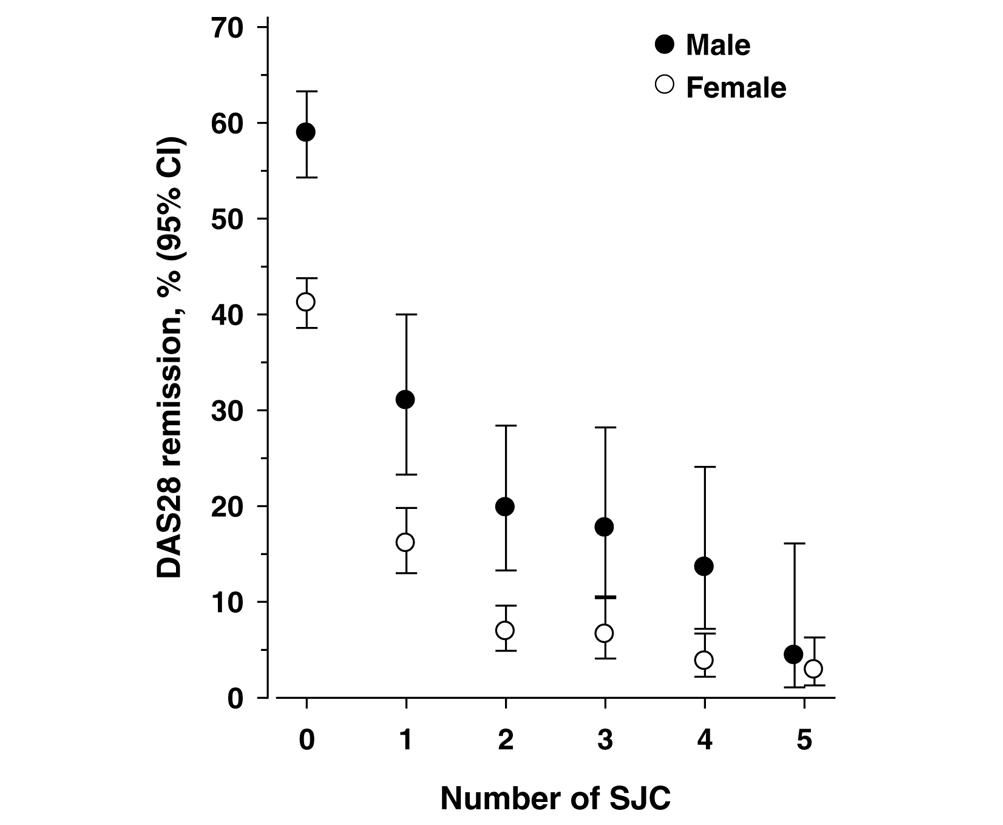
**The proportion of males and females with 0 to 5 swollen joints in the QUEST-RA Study who meet DAS28 criteria for remission**. CI, confidence interval; DAS28, disease activity score using 28 joint counts; QUEST-RA, Quantitative Standard Monitoring of Rheumatoid Arthritis; SJC, swollen joint count.

More men (30.0%) than women (16.7%) were in DAS28 remission (*P *< 0.001). Among patients with 0 swollen joints, 57.6% of men and 42.0% of women were in DAS28 remission. Among patients with 1 and 2 swollen joints, 30.3% and 20.2% of men and 16.9% and 7.1% of women met DAS28 remission, respectively (*P *< 0.001) (Figure 2). Around 15% of men and 5% of women met criteria for DAS28 remission even when they had 3 to 4 swollen joints on a 28-joint count.

### Gender differences in disease activity measures according to country

Standardized units of difference between genders were calculated for each variable according to country and are shown as an example for two variables: HAQ (Table 3) and DAS28 (Table 4). Differences according to gender were greatest on the HAQ (of all Core Data Set measures); females had poorer scores compared with men in all but one country. The effect sizes of gender were high (Cohen's D > 0.5) in 5 countries, medium (0.2 to 0.5) in 16 out of 25 countries, and low (<0.2) in 4 countries (Table 3). For DAS28, the differences between scores according to gender were high in 2 countries, medium in 9 countries, and low in 14 out of 25 countries (Table 4).

**Table 3:** Health Assessment Questionnaire: differences between females and males by country

	Female, mean (SD)	Male, mean (SD)	Difference, mean (95% CI)	Effect size^a^ (95% CI)
Netherlands	0.89 (0.69)	0.62 (0.61)	0.27 (0.12 to 0.43)	0.42 (0.16 to 0.62)
Finland	0.84 (0.76)	0.60 (0.64)	0.24 (0.06 to 0.42)	0.33 (0.07 to 0.56)
USA	0.81 (0.68)	0.52 (0.67)	0.29 (0.12 to 0.46)	0.43 (0.16 to 0.67)
Greece	0.61 (0.71)	0.29 (0.45)	0.32 (0.15 to 0.50)	0.49 (0.29 to 0.68)
Denmark	0.85 (0.75)	0.59 (0.66)	0.26 (0.07 to 0.46)	0.36 (0.09 to 0.60)
Spain	1.10 (0.84)	0.75 (0.69)	0.35 (0.14 to 0.56)	0.44 (0.21 to 0.70)
France	0.99 (0.69)	0.67 (0.62)	0.32 (0.16 to 0.48)	0.48 (0.24 to 0.74)
Sweden	0.97 (0.65)	0.82 (0.63)	0.16 (-0.02 to 0.33)	0.25 (-0.02 to 0.54)
UK	1.06 (0.74)	0.80 (0.75)	0.26 (-0.04 to 0.55)	0.35 (-0.08 to 0.78)
Ireland	0.98 (0.71)	0.79 (0.75)	0.19 (-0.01 to 0.38)	0.26 (-0.04 to 0.55)
Canada	1.04 (0.72)	1.07 (0.73)	-0.03 (-0.40 to 0.34)	-0.04 (-0.58 to 0.44)
Turkey	1.01 (0.77)	0.70 (0.71)	0.31 (0.07 to 0.56)	0.41 (0.11 to 0.73)
Brazil	0.84 (0.74)	0.68 (0.72)	0.15 (-0.28 to 0.59)	0.21 (-0.45 to 0.72)
UAE	0.74 (0.63)	0.42 (0.46)	0.32 (0.07 to 0.56)	0.53 (0.19 to 0.81)
Germany	0.94 (0.70)	0.74 (0.65)	0.20 (-0.04 to 0.45)	0.29 (-0.04 to 0.65)
Italy	1.23 (0.82)	0.72 (0.71)	0.51 (0.30 to 0.71)	0.64 (0.40 to 0.89)
Estonia	1.18 (0.74)	1.08 (0.76)	0.11 (-0.22 to 0.43)	0.14 (-0.29 to 0.59)
Russia	1.24 (0.73)	1.33 (0.59)	-0.09 (-0.56 to 0.37)	-0.13 (-0.77 to 0.43)
Hungary	1.45 (0.66)	0.97 (0.73)	0.48 (0.15 to 0.82)	0.72 (0.08 to 1.23)
Latvia	1.52 (0.66)	1.14 (0.64)	0.38 (0.01 to 0.74)	0.58 (0.03 to 1.16)
Poland	1.40 (0.79)	1.27 (0.68)	0.14 (-0.04 to 0.31)	0.17 (-0.04 to 0.38)
Argentina	1.18 (0.86)	0.97 (0.66)	0.22 (-0.14 to 0.57)	0.26 (-0.11 to 0.58)
Lithuania	1.41 (0.67)	1.21 (0.59)	0.20 (0.00 to 0.40)	0.31 (0.04 to 0.59)
Serbia	1.63 (0.71)	1.24 (0.80)	0.40 (-0.04 to 0.84)	0.55 (-0.24 to 1.18)
Kosovo	1.58 (0.53)	1.34 (0.68)	0.25 (-0.06 to 0.55)	0.44 (-0.13 to 1.15)

All	1.09 (0.78)	0.76 (0.70)	0.33 (0.28 to 0.38)	0.43 (0.37 to 0.49)

^a^Cohen's D with bias-corrected 95% confidence intervals (CIs) from bootstrapping (1,000 replications). SD, standard deviation; UAE, United Arab Emirates.

**Table 4:** DAS28: differences between females and males by country

	Female, mean (SD)	Male, mean (SD)	Difference, mean (95% CI)	Effect size^a^ (95% CI)
Netherlands	3.07 (1.19)	3.03 (1.37)	0.03 (-0.26 to 0.33)	0.03 (-0.23 to 0.29)
Finland	3.37 (1.35)	3.01 (1.60)	0.37 (0.01 to 0.73)	0.26 (-0.03 to 0.55)
USA	3.59 (1.56)	2.65 (1.61)	0.94 (0.54 to 1.35)	0.60 (0.31 to 0.85)
Greece	3.52 (1.49)	2.72 (1.37)	0.79 (0.40 to 1.19)	0.55 (0.28 to 0.80)
Denmark	3.49 (1.41)	3.05 (1.53)	0.44 (0.05 to 0.83)	0.31 (0.03 to 0.60)
Spain	3.59 (1.38)	3.33 (1.31)	0.26 (-0.10 to 0.62)	0.19 (-0.07 to 0.45)
France	3.69 (1.41)	3.56 (1.74)	0.13 (-0.24 to 0.49)	0.09 (-0.22 to 0.36)
Sweden	3.83 (1.58)	3.80 (1.62)	0.04 (-0.40 to 0.48)	0.02 (-0.24 to 0.30)
UK	4.09 (1.41)	3.56 (1.34)	0.53 (-0.04 to 1.11)	0.39 (-0.05 to 0.74)
Ireland	4.16 (1.51)	4.03 (1.86)	0.13 (-0.32 to 0.57)	0.08 (-0.25 to 0.34)
Canada	4.13 (1.57)	4.17 (1.88)	-0.04 (-0.88 to 0.79)	-0.03 (-0.60 to 0.57)
Turkey	4.16 (1.43)	4.12 (1.27)	0.05 (-0.44 to 0.54)	0.03 (-0.28 to 0.37)
Brazil	4.25 (1.41)	3.66 (1.52)	0.59 (-0.25 to 1.42)	0.42 (-0.20 to 1.08)
UAE	4.33 (1.72)	3.83 (1.90)	0.50 (-0.25 to 1.24)	0.29 (-0.20 to 0.72)
Germany	4.47 (1.63)	3.84 (1.97)	0.64 (0.03 to 1.25)	0.38 (-0.00 to 0.79)
Italy	4.51 (1.24)	4.38 (1.37)	0.13 (-0.22 to 0.48)	0.10 (-0.17 to 0.42)
Estonia	4.70 (1.43)	4.53 (1.83)	0.16 (-0.50 to 0.83)	0.11 (-0.37 to 0.63)
Russia	4.89 (1.46)	5.33 (0.86)	-0.44 (-1.40 to 0.51)	-0.32 (-0.82 to 0.19)
Hungary	5.13 (1.23)	4.51 (1.24)	0.61 (0.00 to 1.23)	0.50 (0.02 to 1.07)
Latvia	5.24 (1.59)	5.11(1.41)	0.13 (-0.78 to 1.03)	0.08 (-0.49 to 0.68)
Poland	5.32 (1.44)	5.20 (1.43)	0.12 (-0.22 to 0.45)	0.08 (-0.15 to 0.32)
Argentina	5.35 (1.68)	5.36 (1.95)	-0.02 (-0.76 to 0.72)	-0.01 (-0.48 to 0.54)
Lithuania	5.49 (1.30)	5.51 (1.30)	-0.02 (-0.43 to 0.38)	-0.02 (-0.34 to 0.30)
Serbia	5.93 (1.30)	5.93 (0.97)	-0.01 (-0.78 to 0.77)	-0.00 (-0.45 to 0.51)
Kosovo	6.08 (0.95)	5.83 (0.99)	0.25 (-0.26 to 0.77)	0.27 (-0.32 to 0.91)

All	4.30 (1.64)	3.76 (1.76)	0.54 (0.43 to 0.65)	0.33 (0.25 to 0.39)

^a^Cohen's D with bias-corrected 95% confidence intervals (CIs) from bootstrapping (1,000 replications). DAS28, disease activity score using 28 joint counts; SD, standard deviation; UAE, United Arab Emirates.

### Gender and disease characteristics

RF was equally prevalent among females and males, including 'low' (females 67.2% versus males 69.5%; *P *= 0.29) and 'high' (79.6% versus 80.0%; *P *= 0.86) prevalence countries. Men (24.1%) had rheumatoid nodules more often than women (19.3%). Erosions were more prevalent among women than men (64.3% versus 59.7%; *P *= 0.003), although the difference was not statistically significant in 'low' prevalence countries (53.6% versus 51.6%; *P *= 0.36) and was only marginally significant in 'high' prevalence countries (76.7% versus 71.9%; *P *= 0.041). Men were smokers more often than women: 27.2% versus 14.8% (*P *< 0.001).

### Gender and therapies for rheumatoid arthritis

In 'low use' countries, similar percentages of women and men were currently taking prednisone (30.0% versus 31.0%), methotrexate (54.1% versus 54.7%), and biologic agents (7.9% versus 8.1%). Similar proportions of women and men were taking these drugs in 'high use' countries; the percentages were 60.6% versus 61.5% for prednisone, 68.4% versus 72.0% for methotrexate, and 29.1% versus 30.8% for biologic agents, respectively. Similar percentages of women and men had ever taken these drugs over the course of RA (data not shown). The delay between the first RA symptoms and initiation of DMARDs was 10 months in the entire group, with considerable variation between countries (Table 1); no statistically significant gender differences within countries were seen (data not shown).

## Discussion

Obvious differences between genders exist in the prevalence, age at onset, and autoantibody production of RA [39]. The majority of patients with RA are middle-aged women, generally greater than 70% in any RA cohort (including the present study), although RA can occur at any age in either gender. Furthermore, gender differences are seen in biologic (hormones) [40] and behavioral (smoking) [41,42] factors that may influence susceptibility and phenotype of RA.

As noted before, the natural history of RA when limited treatment options were available involved severe outcomes in most patients without major gender differences. However, occasional reports of gender differences in RA with unexpected results or interpretations appear to have gained more attention than reports of no gender differences. Ten years ago, a study from the Mayo Clinic [18] compared 55 male patients with 110 female controls with similar disease duration of at least 10 years. Erosive disease was more prevalent and developed earlier in men than in women. Nodules and lung disease were more frequent in men and sicca syndrome was more frequent in women [18]. The investigators suggested that the findings might help 'assess the prognosis and tailor the treatment of the individual patient' with a reaction from the rheumatology community [1].

Our results are consistent with the results of recent studies that indicate major gender differences in DAS28 remission rates: overall, 30% of men and 17% of women in QUEST-RA were in DAS28 remission. Differences were striking in patients who had no swollen joints: much fewer women than men (42% versus 58%) met DAS28 remission. At SJC28 levels of 0 to 1, which indicate no or very little clinical disease activity, gender differences were significant both clinically and statistically in all other American College of Rheumatology (ACR) Core Data Set measures and fatigue. On the other hand, gender differences in measures were less pronounced or nonexistent on higher disease activity levels (that is, higher SJC28 counts) (Table 2). Therefore, recent observations of higher DAS28 remission rates [22,23] and treatment responses in males [19-21] may reflect considerable differences in the measures between genders [27,43-45], including normal ESR levels, which are higher in females than males (especially in older age groups) [28]. Furthermore, women report more symptoms and poorer scores on most questionnaires [7], including scores for pain [46], depression, and other health-related items [47,48].

Self-report performance in activities of daily living is a strong predictor of further functional loss, work disability, and mortality in RA, in other conditions, and in the general population [49]. HAQ is an important outcome measure in clinical trials and in the documentation of patient status in clinical care [33]. Throughout the history of the HAQ, women have been found to report poorer scores than men [8,50-53]. This is reasonable as women are not as physically strong as men [54,55], which has a major effect in the functional status of patients with RA and of healthy persons [56]. In fact, gender differences in musculoskeletal performance remain even among the best-trained individuals – female and male athletes compete separately! Given that women are a 'weaker vessel' concerning musculoskeletal size and strength and their baseline values are lower than those of men, the same burden of a musculoskeletal disease may be more harmful to a woman than to a man.

Possible reasons for gender differences in RA have been sought on the basis of sex hormones. Disease activity is ameliorated in 75% of women in pregnancy, and after delivery, flares occur in up to 90% [57]. Oral contraceptives may protect against RA [58]. Hormone replacement therapy appears to be beneficial concerning RA disease activity [59]. Estrogen has a dichotomous impact on the immune system by downregulating inflammatory immune responses and upregulating immunoglobulin production [60]. On the other hand, sex hormone metabolism in RA synovial tissues may be unfavorable for females; tumor necrosis factor inhibitors alter sex hormone metabolism in the synovial tissue [61]. The beneficial effects include restored levels of synovial androgens although restored androgenic (immunosuppressive) activity may explain, in part, the higher likelihood of men to develop serious infections during biologic treatments [24,25].

Radiographs provide a permanent measure of the structural damage of RA, radiographic scores are associated with certain disease activity measures [62]. Gossec and colleagues [63] found no statistically significant differences in radiographic outcomes between genders. In the BARFOT (Better Anti-rheumatic Farmacotherapy) study [64] of patients with early RA, erosive disease was present in 27% of men and 28% of women at the time of diagnosis. Similar percentages of females and males were free of any radiographic changes over the course of 2 years [64], and radiographic scores remained similar between genders during the follow-up of 5 years [44]. In the extensive database of Wolfe and Sharp [65] concerning number of patients and number of years, gender was not among predictors of radiographic progression over the course of two decades. These observations are consistent with early studies from the 1980s [66] indicating that RA presents similarly in both genders in case the extent of structural damage is chosen as the measure of disease severity.

There is a concern that women might be less likely to be treated aggressively for RA compared with men. A report from The Netherlands indicates a longer delay of referral of females to an early arthritis clinic compared with men [67]. Several reports from the cardiology literature indicate that men are treated more intensively than women [68,69]. We did not find significant differences in the proportion of females and males who were taking prednisone, methotrexate, and biologic agents in the QUEST-RA Study. Furthermore, the delay to initiation of therapies was similar for females and males within countries.

Although QUEST-RA represents a unique program, several limitations are recognized. All data were collected as part of clinical care in different clinical environments and traditions to examine and treat patients, which may vary greatly in the participating countries. First, a central laboratory was not used for blood samples, which instead were analyzed locally. Therefore, for example, normal CRP was reported as '<10' in many clinics and DAS28-CRP cannot be calculated for all patients; CRP values of 0 to 9.9 provide DAS28 results different from CRP = 10, especially on low DAS28 levels approaching criteria for remission. Second, although radiographs were taken of most patients, they were analyzed by treating rheumatologists for erosive or nonerosive disease only, and quantitative scoring was not performed. Third, a cross-sectional database may not be ideal to study gender differences, and longitudinal observations might provide a more accurate picture of gender differences in RA, with follow-up of all long-term outcomes, including mortality. Men tend to die earlier than women and may therefore be 'left-censored' in cross-sectional databases, rendering outcomes for men apparently better. Finally, the QUEST-RA data may not be generalizable in all included (or nonincluded) countries.

## Conclusion

QUEST-RA data indicate that currently used disease activity measures are higher in women than in men. Gender differences for DAS28, fatigue, and ACR Core Data Set measures are most pronounced in patients with low swollen joint counts, suggesting that (especially at low levels of disease activity) one has to be cautious about interpretations of gender differences since disease activity measures themselves may be contaminated by gender.

## Abbreviations

ACR: American College of Rheumatology; CRP: C-reactive protein; DAS28: disease activity score using 28 joint counts; DMARD: disease-modifying antirheumatic drug; ESR: erythrocyte sedimentation rate; HAQ: Health Assessment Questionnaire; QUEST-RA: Quantitative Standard Monitoring of Rheumatoid Arthritis; RA: rheumatoid arthritis; RF: rheumatoid factor; SJC28: swollen joint count-28; TJC28: tender joint count-28.

## Competing interests

One or more authors of this manuscript have received reimbursements, fees, or funding from pharmaceutical companies. The article-processing charge was not received from any of these companies: Abbott (Abbott Park, IL, USA), Allergan (Irvine, CA, USA), Amgen (Thousand Oaks, CA, USA), Bristol-Myers Squibb Company (Princeton, NJ, USA), Chelsea Therapeutics (Charlotte, NC, USA), GlaxoSmithKline (Uxbridge, Middlesex, UK), Jazz Pharmaceuticals (Palo Alto, CA, USA), Merrimack Pharmaceuticals (Cambridge, MA, USA), MSD (Whitehouse Station, NJ, USA), Pfizer Inc (New York, NY, USA), Pierre Fabre Médicament (Boulogne Cedex, France), Roche (Basel, Switzerland), Schering-Plough Corporation (Kenilworth, NJ, USA), sanofi-aventis (Paris, France), UCB (Brussels, Belgium), and Wyeth (Madison, NJ, USA).

## Authors' contributions

The QUEST-RA Study was designed and conducted by TS and TPincus. All authors participated in data collection concerning their clinical patients. Analyses for the present report were designed and coordinated by TS and HK. All authors read and approved the final manuscript.

## Authors' information

The QUEST-RA Group is composed of the following members: **Argentina**: Sergio Toloza, Santiago Aguero, Sergio Orellana Barrera, Soledad Retamozo, Hospital San Juan Bautista, Catamarca; Paula Alba, Cruz Lascano, Alejandra Babini, Eduardo Albiero, Hospital of Cordoba, Cordoba; **Brazil**: Geraldo da Rocha Castelar Pinheiro, Universidade do Estado do Rio de Janeiro, Rio de Janeiro; Licia Maria Henrique da Mota, Hospital Universitário de Brasília; Ines Guimaraes da Silveira, Pontifícia Universidade Católica do Rio Grande do Sul (PUCRS), Porto Alegre; Francisco Airton Rocha, Universidade Federal do Ceará, Fortaleza; Ieda Maria Magalhães Laurindo, Universidade Estadual de São Paulo, São Paulo; **Canada**: Juris Lazovskis, Riverside Professional Center, Sydney, NS; **Denmark**: Merete Lund Hetland, Lykke Ørnbjerg, Copenhagen Univ Hospital at Hvidovre, Hvidovre; Kim Hørslev-Petersen, King Christian the Xth Hospital, Gråsten; Troels Mørk Hansen, Lene Surland Knudsen, Copenhagen University Hospital at Herlev, Herlev; **Estonia**: Raili Müller, Reet Kuuse, Marika Tammaru, Riina Kallikorm, Tartu University Hospital, Tartu; Tony Peets, East-Tallinn Central Hospital, Tallinn; Ivo Valter, Center for Clinical and Basic Research, Tallinn; **Finland**: Heidi Mäkinen, Jyväskylä Central Hospital, Jyväskylä; Kai Immonen, Sinikka Forsberg, Jukka Lähteenmäki, North Karelia Central Hospital, Joensuu; Reijo Luukkainen, Satakunta Central Hospital, Rauma; **France**: Laure Gossec, Maxime Dougados, University René Descartes, Hôpital Cochin, Paris; Jean Francis Maillefert, Dijon University Hospital, University of Burgundy, Dijon; Bernard Combe, Hôpital Lapeyronie, Montpellier; Jean Sibilia, Hôpital Hautepierre, Strasbourg; **Germany**: Gertraud Herborn, Rolf Rau, Evangelisches Fachkrankenhaus, Ratingen; Rieke Alten, Christof Pohl, Schlosspark-Klinik, Berlin; Gerd R Burmester, Bettina Marsmann, Charite-University Medicine Berlin, Berlin; **Greece**: Alexandros A Drosos, Sofia Exarchou, University of Ioannina, Ioannina; H M Moutsopoulos, Afrodite Tsirogianni, School of Medicine, National University of Athens, Athens; Fotini N Skopouli, Maria Mavrommati, Euroclinic Hospital, Athens; **Hungary**: Pál Géher, Semmelweis University of Medical Sciences, Budapest; Bernadette Rojkovich, Ilona Újfalussy, Polyclinic of the Hospitaller Brothers of St. John of God in Budapest, Budapest; **Ireland**: Barry Bresnihan, St. Vincent's University Hospital, Dublin; Patricia Minnock, Our Lady's Hospice, Dublin; Eithne Murphy, Claire Sheehy, Edel Quirke, Connolly Hospital, Dublin; Joe Devlin, Shafeeq Alraqi, Waterford Regional Hospital, Waterford; **Italy**: Massimiliano Cazzato, Stefano Bombardieri, Santa Chiara Hospital, Pisa; Gianfranco Ferraccioli, Alessia Morelli, Catholic University of Sacred Heart, Rome; Maurizio Cutolo, University of Genova, Genova, Italy; Fausto Salaffi, Andrea Stancati, University of Ancona, Ancona; **Kosovo**: Sylejman Rexhepi, Mjellma Rexhepi, Rheumatology Department, Pristine; **Latvia**: Daina Andersone, Pauls Stradina Clinical University Hospital, Riga; **Lithuania**: Sigita Stropuviene, Jolanta Dadoniene, Institute of Experimental and Clinical Medicine at Vilnius University, Vilnius; Asta Baranauskaite, Kaunas University Hospital, Kaunas; **The Netherlands**: Suzan MM Verstappen, Johannes WG Jacobs, University Medical Center Utrecht, Utrecht; Margriet Huisman, Sint Franciscus Gasthuis Hospital, Rotterdam; Monique Hoekstra, Medisch Spectrum Twente, Enschede; **Poland**: Stanislaw Sierakowski, Medical University in Bialystok, Bialystok; Maria Majdan, Medical University of Lublin, Lublin; Wojciech Romanowski, Poznan Rheumatology Center in Srem, Srem; Witold Tlustochowicz, Military Institute of Medicine, Warsaw; Danuta Kapolka, Silesian Hospital for Rheumatology and Rehabilitation in Ustron Slaski, Ustroñ Slaski; Stefan Sadkiewicz, Szpital Wojewodzki im. Jana Biziela, Bydgoszcz; Danuta Zarowny-Wierzbinska, Wojewodzki Zespol Reumatologiczny im. dr Jadwigi Titz-Kosko, Sopot; **Russia**: Dmitry Karateev, Elena Luchikhina, Institute of Rheumatology of Russian Academy of Medical Sciences, Moscow; Natalia Chichasova, Moscow Medical Academy, Moscow; Vladimir Badokin, Russian Medical Academy of Postgraduate Education, Moscow; **Serbia**: Vlado Skakic, Aleksander Dimic, Jovan Nedovic, Aleksandra Stankovic, Rheumatology Institut, Niska Banja; **Spain**: Antonio Naranjo, Carlos Rodríguez-Lozano, Hospital de Gran Canaria Dr. Negrin, Las Palmas; Jaime Calvo-Alen, Hospital Sierrallana Ganzo, Torrelavega; Miguel Belmonte, Hospital General de Castellón, Castellón; **Sweden**: Eva Baecklund, Dan Henrohn, Uppsala University Hospital, Uppsala; Rolf Oding, Margareth Liveborn, Centrallasarettet, Västerås; Ann-Carin Holmqvist, Hudiksvall Medical Clinic, Hudiksvall; **Turkey**: Feride Gogus, Gazi Medical School, Ankara; Recep Tunc, Meram Medical Faculty, Konya; Selda Celic, Cerrahpasa Medic Faculty, Istanbul; **United Arab Emirates**: Humeira Badsha, Dubai Bone and Joint Center, Dubai; Ayman Mofti, American Hospital Dubai, Dubai; **UK**: Peter Taylor, Catherine McClinton, Charing Cross Hospital, London; Anthony Woolf, Ginny Chorghade, Royal Cornwall Hospital, Truro; Ernest Choy, Stephen Kelly, Kings College Hospital, London; **USA**: Theodore Pincus, Vanderbilt University, Nashville, TN; Yusuf Yazici, NYU Hospital for Joint Diseases, New York, NY; Martin Bergman, Taylor Hospital, Ridley Park, PA; Christopher Swearingen, Medical University of South Carolina, Charleston, SC; **Study Center**: Tuulikki Sokka, Jyväskylä Central Hospital, Jyväskylä, Medcare Oy, Äänekoski, Finland; Hannu Kautiainen, Medcare Oy, Äänekoski, Finland; Theodore Pincus, New York University Hospital for Joint Diseases, New York, NY, USA.
